# Tactile, Audio, and Visual Dataset During Bare Finger Interaction with Textured Surfaces

**DOI:** 10.1038/s41597-025-04670-0

**Published:** 2025-03-23

**Authors:** Alexis W. M. Devillard, Aruna Ramasamy, Xiaoxiao Cheng, Damien Faux, Etienne Burdet

**Affiliations:** 1 Imperial College of Science, Technology and Medicine, London, W12 0BZ UK; 2 École Normale Supérieure, CNRS, Laboratoire des Systèmes Perceptifs, Paris, 75005 France; 3 University of Manchester, Manchester, M139PL UK; 4 Actronika, Paris, 93500 France

**Keywords:** Biomedical engineering, Scientific data

## Abstract

This paper presents a comprehensive multi-modal dataset capturing concurrent haptic, audio, and visual signals recorded from ten participants as they interacted with ten different textured surfaces using their bare fingers. The dataset includes stereoscopic images of the textures, and fingertip position, speed, applied load, emitted sound, and friction-induced vibrations, providing an unprecedented insight into the complex dynamics underlying human tactile perception. Our approach utilizes a human finger (while most previous studies relied on rigid sensorized probes), enabling the naturalistic acquisition of haptic data and addressing a significant gap in resources for studies of human tactile exploration, perceptual mechanisms, and artificial tactile perception. Additionally, fifteen participants completed a questionnaire to evaluate their subjective perception of the surfaces. Through carefully designed data collection protocols, encompassing both controlled and free exploration scenarios, this dataset offers a rich resource for studying human multi-sensory integration and supports the development of algorithms for texture recognition based on multi-modal inputs. A preliminary analysis demonstrates the dataset’s potential, as classifiers trained on different combinations of data modalities show promising accuracy in surface identification, highlighting its value for advancing research in multi-sensory perception and the development of human-machine interfaces.

## Background & Summary

Human perception is inherently multi-sensory, involving the integration of information from various modalities to enhance the robustness and accuracy of our understanding of environmental characteristics. This integration is crucial for resolving ambiguities, ensuring perceptual stability, and improving the perception of object properties under uncertain conditions^1–4^. Touch, audition and vision are the primary senses that contribute to perceiving the physical properties of surfaces. Stereoscopic visual data, for example, provides depth information essential for understanding a surface’s topography and texture^5^. Auditory cues, particularly those produced by mechanical interactions such as sliding contacts, serve as vital sources of perceptual information^6,7^. Additionally, touch is a complex sense that integrates various sub-modalities, including force, vibration, and proprioception^8,9^. The interdependence of these modalities is evident in phenomena such as the pseudo-haptic effect, where visual stimuli create an illusion of haptic feedback^10^, and the influence of auditory cues on the perception of hand dryness^11^. Therefore, datasets encompassing these sensory modalities are essential for advancing perceptual studies and developing artificial sensory feedback systems that fully exploit the multi-sensory nature of human perception.

Previous research has primarily focused on capturing images of textures and associated haptic signals, such as acceleration or load, using rigid probes^12–17^. Some studies have also investigated the auditory aspects of sliding interactions^18,19^. These efforts have provided valuable insights into the role of individual sensory modalities in texture perception, facilitating the development of haptic rendering systems integrated into simple styluses or robotic fingers^20^. However, most of these studies relied on rigid, sensorized probes and did not capture the direct interaction of a bare human finger with surfaces. Other research has documented the load and vibrations on a substrate from a bare finger sliding on a textured surface^21,22^ or the skin vibrations during sliding^23,24^.

Finally a critical feature for a comprehensive dataset is the presence of a subjective evaluation of the surfaces. This evaluation is essential to understand how the physical properties of the surfaces are perceived by humans. Recent work such as the sens3^25^ presents a multi-sensory dataset (position, audio, vibration and temperature) recorded on two participants and 50 textures coupled with subjective evaluations. Sens3 employs an ADXL356 accelerometer with a bandwidth of 2.6 kHz, which is sufficient to capture vibrations above the threshold of human tactile perception^26^. However, as the dataset is recorded at 10 kHz, a substantial portion of the acquired signal falls outside the sensor’s effective range, limiting its usefulness for analysing the mechanical propagation of high-frequency vibrations through the skin^27^. Additionally, the dataset lacks precise sensor synchronization, thereby limiting its applicability for high-frequency signal analysis. Existing datasets with their key features are summarized in Table 1.

**Table 1:** Summary of existing multi-sensory datasets capturing tactile interactions.

Name	Modalities	Interaction	Instrument	Questionnaire	Availability
^21^	Force and vibration	Slide	Bare finger	No	Private
HaTT^13^	Position, force, vibration and images	Slide	Sensorised probe	No	Public
^14^	Position, force, vibration and images	Teleoperation	Phantom haptic device	No	Private
^22^	Position, Load and frictional forces	Slide	Bare finger	No	Private
^15^	Position, force, vibration and images	Slide	Sensorised probe	No	Private
^18^	Audio	Slide	Human finger	No	Private
CBSMR^16^	Position, force, vibration and images	Slide	Sensorised probe	Yes	Depreciated
HapTex^39^	Position, force, vibration and images	Slide	Guided bare finger	No	Private
^40^	Vibration and images	Slide	bare finger	No	Private
LMT^17^	Position, force, vibration audio and images	Slide	Sensorised probe + Bare finger	No	Public
Viper^19^	Images and vibration	Slide	Sensorised probe	Yes	Public
Sens3^25^	Position, force, vibration, audio, temperature and images	Slide, press, tap	Bare finger	Yes	Public

To our knowledge, no comprehensive dataset exists that integrates synchronized visual, audio, and haptic signals collected from finger exploration of material surfaces, representing a significant gap in resources for perceptual studies and machine learning. Such a dataset would enable the study of the complex interplay between different sensory modalities in human tactile perception and could serve as a foundation for inducing haptic feedback directly on the human finger^28^, as well as for training machine learning algorithms to recognize textures based on multi-modal inputs.

This gap motivated us to create a multi-modal dataset by collecting signals measured when a bare finger interacts with various textured surfaces. The dataset includes stereoscopic images of the surface, fingertip position and speed in image coordinates, the applied load by the finger, emitted sound, and friction-induced vibrations propagating through the finger. While the use of an artificial finger was considered^29–31^, we opted for bare fingers to ensure natural data collection.

The potential of this dataset is demonstrated by exploring the accuracy of classifiers trained on different combinations of these multi-modal data to identify surfaces. The following sections detail the data acquisition apparatus and protocol, the surfaces used (Section Methods), the dataset’s structure (Section Data Records), and the technical validation of the dataset (Section Technical Validation).

This dataset addresses a critical gap in multi-sensory perception research and opens new avenues for advancements in perception studies, offering unprecedented insights into the nuanced interplay of touch, vision, and sound in human perception.

## Methods

### Experimental setup

**Fig. 1:**
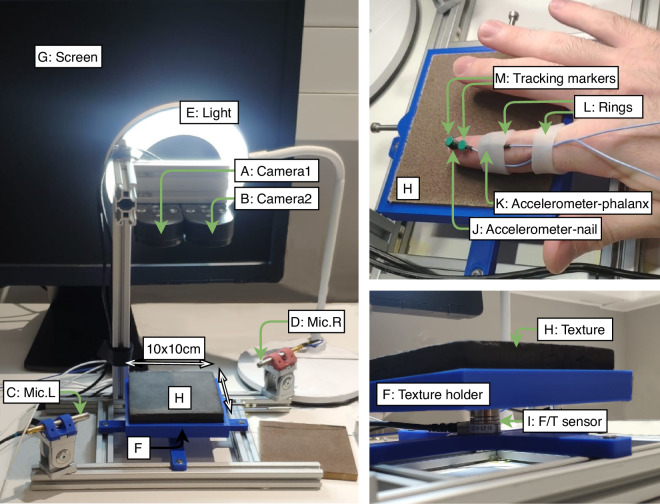
Experimental setup used to collect multi-modal data from human fingertip interactions with various textured surfaces. (A,B) 4K cameras 1 and 2 above a textured surface. (C,D) Directional microphones Left and Right with adjustable support. (E) Light source. (F) Texture holder. (G) Screen to display GUI. (H) Texture. (I) Force/torque sensor. (J) Accelerometer-nail glued to the nail, and (K) accelerometer phalanx strapped to the index finger by a (L) Silicone ring. (K) 2 Tracking markers for the fingertip motion and direction recording.

The dedicated setup shown in Fig. 1, installed in a sound-proofed room to minimize external noise interference, was designed to record high-resolution visual, audio, and haptic data as a participant’s finger slides against differently textured surfaces. It consists of the following components:

#### Visual data acquisition

**Fig. 2:**
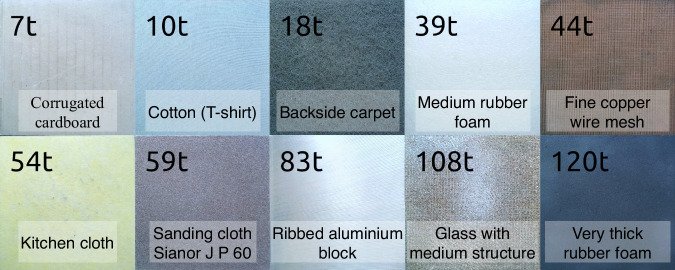
Representative textures used in the experiments, selected from the larger set of study^33^.

To study and understand the role of visual cues in haptic perception, we recorded high-resolution stereoscopic images of the textured surfaces (Fig. 2). Similar to human vision, stereoscopic images provide depth information, which is crucial for understanding the surface’s topography and texture:

- **Vision**. The visual data acquisition setup consisted of two 4K cameras (Fig. 1A,B, model QXC-700, Thustar, Dongguan, China) equipped with close-range lenses to record the textured surfaces. The cameras were positioned 5 cm apart, capturing the surfaces from slightly different angles. Each surface was photographed at a resolution of 3840 × 2160.

#### Tactile data acquisition

To capture the complexity and multi-dimensionality of tactile interactions, we used several type of sensors to record the force, vibrations, and position of the finger during the interaction:

- **Force/torque**. The load applied by the finger on the surface impacts the physical interaction and the perception of the texture. This load was recorded using a six-axis force/torque (F/T) sensor (Fig. 1I : Model Nano17-SI-25-0.25, ATI Industrial Automation, Apex, NC, USA) with a resolution of 1/160 N and 1/32 N mm mounted on a 3D-printed holder attached to the aluminium profile structure. The sensor was positioned 5 cm below the texture surface.
- **Vibrations**. When the fingertip is sliding on a textured surface, vibrations are generated and propagate through the finger. We recorded these vibrations using two accelerometers (Fig. 1J,K: Model 8778A500, Kistler Instrumente AG, Winterthur, Switzerland) with a sensitivity of 10 mV/g and a frequency range from 2 to 9000 Hz. To capture these vibrations the position and fixation of the accelerometers are critical. The first accelerometer was attached to the finger’s nail with glue to ensure a rigid fixation and prevent damping effects. The second accelerometer was attached to the finger’s first phalanx to evaluate the vibrations propagating through the finger. Due to the soft nature of the finger skin, this second accelerometer could not be rigidly fixed, instead we used a silicone ring to ensure a comfortable fixation. The data recorded from these accelerometers will then be referred to as ‘Vibration-nail’ and ‘Vibration-phalanx’ respectively.
- **Fingertip position and speed**. To understand the impact of the sliding speed and direction on the gathered data, we recorded the position and speed of the fingertip. We used two tracking markers (Fig. 1M) on the fingernail to compute the centroid of the fingertip on the surface.

#### Audio data acquisition

Sound produced by the interaction between the finger and each textured surfaces was also recorded to capture any auditory cues generated. The sound produced by the finger-surface interaction has a low amplitude relative to the background noise (e.g., the sound of the participant’s breathing):

- **Audio**. To focus on the sound generated by the interaction, we used two directional microphones(Fig. 1C,D; Model 130A23, PCB Piezotronics Inc., Depew, NY, USA) with a sensitivity of 14 mV/Pa. The microphones were place pointing towards the finger and the surface in two opposite directions in order to facilitate audio post-processing and noise reduction.

#### Signal conditioning and synchronization

**Fig. 3:**
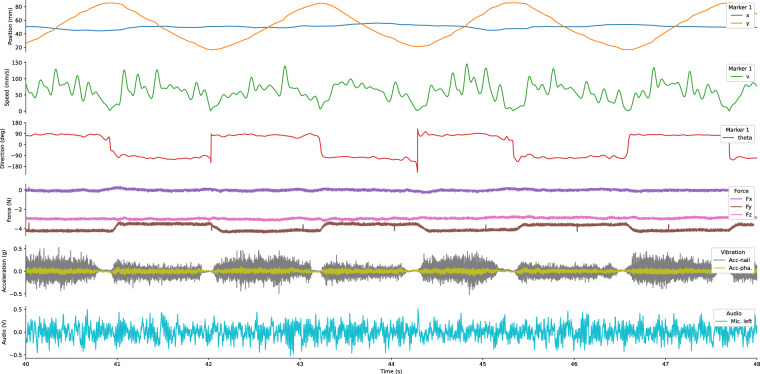
Visualisation of some of the physical signals recorded during lateral sliding (phase 2.c of a trial). The figure shows the following signals from top to bottom: position, speed and velocity direction of the first marker; force measured below the center of the surface; vibration (acceleration) measured on the nail and phalanx; audio signal from the left microphone.

The quality of the recorded data is highly dependent on the signal conditioning and synchronization of the different data sources. The microphone and accelerometers were connected to a laboratory amplifier and acquisition device (Model 5165A, Kistler Instrumente AG, Winterthur, Switzerland) that recorded the audio and acceleration signals at a 12.5 kHz sampling rate with 32-bit resolution. The synchronization of these signals was at the hardware level by the signal conditioning unit. Additionally, to synchronize every data source, the computer’s kernel clock was utilized to timestamp each received data chunk. This data was streamed using a LabStreamingLayer architecture^32^. Concurrently, a multi-threaded process on the computer collected all incoming data, directing it into a PostgreSQL (PSQL) database. Given the high volume of data produced by audio and acceleration streaming at 12.5 kHz, the TimeScaleDB PostgreSQL extension, designed specifically for efficient time series data storage, was employed to enhance the database’s data storage speed. Fig. 3 illustrates the recorded and processed signals. The dataset is provided with the timestamp of each data point and each sensor’s original sampling rate. For further processing and analysis, the data can be aligned through down- or up-sampling depending on the desired rate.

#### Synchronization strategy

To ensure the synchronization of the data streams, every device was first synchronized to the main computer clock using the NTP protocol, then each chunk of data was timestamped using the computer clock. The dataset is provided with the timestamp of each data point.

#### Texture surfaces

Ten texture surfaces were selected from a larger set^33^ to represent a diverse range of materials and surface topographies. These surfaces are displayed in Fig. 2 and are labeled by the same code as in the original study^33^. To select textures, we explored the samples by sliding our fingers over their surfaces, selecting those expected to generate distinct acoustic and vibratory signatures. Textures that impeded smooth sliding, such as flat metals and glass surfaces, were excluded due to their propensity to induce irregular motion during interaction. Additionally, textures with visual patterns that did not contribute meaningfully to haptic perception (e.g., printed images) were excluded.

#### Visual feedback mechanism

**Fig. 4:**
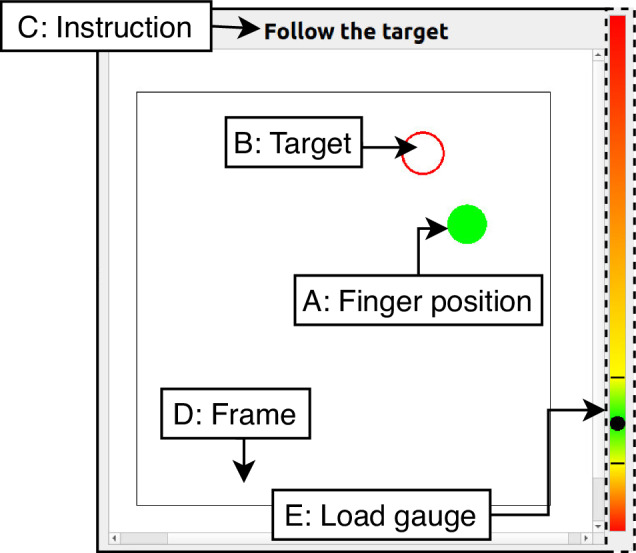
Graphical User Interface (GUI). The interface shows a filled circle (A) indicating fingertip position and an empty square (D) representing the texture’s boundaries. The user instructions are displayed on top of the GUI (C). Depending on the trial type, the GUI can display a moving target (B) and/or a load gauge (E).

During each trial, participants were seated in front of a screen providing visual feedback for fulfilling the trial constraints (see Section Experimental protocol). The screen displayed a filled circle to indicate the relative position of the fingertip on the texture (Fig. 4). An empty square was also shown to display the boundaries of the texture within which the participant was required to stay.

### Experimental protocol

We collected and analyzed multi-modal sensory data from human fingertip interactions with various textured surfaces. The data collection was approved by the Research Ethics Committee of Imperial College London (approval number: 22IC7699). Ten participants, aged between 18 and 50 years (5 males, 5 females) were recruited and provided written informed consent before starting with the experiment. All participants were right-handed and had no known sensory or motor impairments. The recordings were conducted between August and September 2023. The data collection encompassed 50 trials for each participant, with five trials for each of the ten selected textures, and lasted around 1 h.

**Fig. 5:**
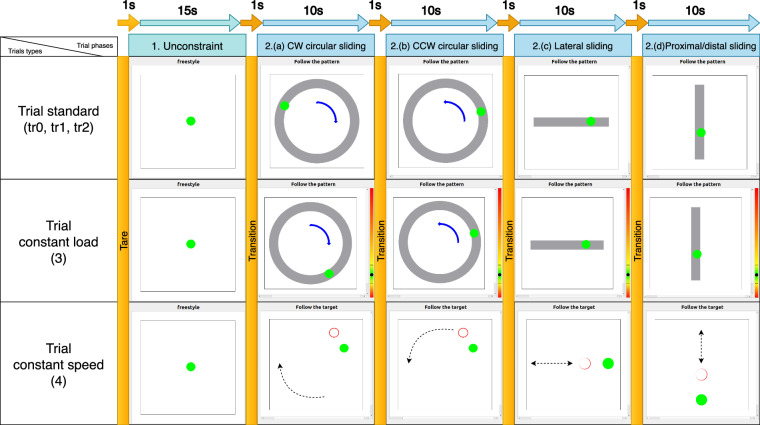
Trials types and associated GUI. Each participant performed 50 trials, divided into three types: Standard, Constant Speed, and Constant Load. Each trial was further divided into five phases: unrestricted sliding, clockwise circular sliding, anti-clockwise circular sliding, lateral back-and-forth sliding, and proximal/distal back-and-forth sliding.

The textures examined in each trial were selected in a randomized order to prevent order effects. At the start of each trial, participants positioned their finger above the texture, allowing for load calibration without making contact. The trials were divided into three types: *Standard*, *Constant Speed*, and *Constant Load*, each with specific constraints. The order of execution of the type of trials was kept constant within each texture. The trials were further divided into five phases, each designed to capture different aspects of tactile interaction (Fig. 5).

#### Trial phases

The trials, each lasting a minute, were segmented into the following four phases designed to obtain rich data (as shown in the columns of Fig. 5):

0. Before the start of a trial, participants place their finger above the texture without contact. The experimenter then starts recording, the GUI displays a “Wait” message for 1s, during which the texture’s weight is automatically calibrated. The task instructions then appear on the display, prompting the participant to begin interacting with the texture.
1. In the first 15 seconds participants can freely move their finger along the surface while varying the load, and adjusting their scanning speed.
2. The next 40 seconds are divided into four 10 second segments, each with a specific constraint:
  - clockwise circular sliding.
  - anti-clockwise circular sliding.
  - lateral back-and-forth sliding.
  - proximal/distal back-and-forth sliding.
3. The trial ends automatically. The participant then removes the finger from the texture, allowing the experimenter to exchange the texture for the next trial.

Each segment included an initial 1-second transition period, ensuring a full 10 seconds period of effective recording for each task.

#### Trial types

The five trials for each texture were divided into three types of trials (corresponding to the rows in Fig. 5), each designed to assess different aspects of tactile interaction:

During the first three trials *Standard*) the speed and load were not constrained. The GUI displayed a static shape (either circular for phases 2a and 2b or rectangular for trial’s phases 2c and2d) positioned at the texture’s centre. Participants were required to move their finger periodically within this shape while maintaining contact with the texture. During the fourth trial (*Constant Load*) participants were asked to maintain a constant load of 4 ± 1 N. The GUI displayed a color gauge indicating the normal load exerted by the participant. The goal was to maintain this load within displayed limits. Finally, during the fifth trial (*Constant Speed*) participants were asked to track a moving target. The GUI displayed a circle moving at a constant speed of 0.1 m/s within the texture’s boundaries (Fig. 4B). The goal was to maintain contact with the moving target. The target’s motion followed a predefined trajectory in accordance with the trial’s phase.

This protocol was designed to capture a comprehensive range of tactile interactions in terms of contact force, sliding speed and movement directions, providing rich data for subsequent analysis in perceptual, artificial perception, and machine learning studies.

### Texture images

To complement the dynamic physical data, ten high-resolution images were captured from each camera per texture. Each image was taken with different lighting configurations to capture the texture’s appearance under various conditions. These images are provided in the dataset to enable the study of the relationship between the physical properties of the surfaces and their visual appearance.

### Psychophysical evaluation

We conducted a psychophysical evaluation to evaluate the subjective perception of the surfaces that complements the physical description of the interactions. This evaluation was designed to capture the participants’ perception of the surfaces in terms of tactile, visual, and auditory aspects. It can be used to study the relationship between the physical properties of the surfaces and the participant’s subjective perceptions.

Fifteen participants different from those of the first experiment were recruited (7 males and 8 females, between 18 and 50 years old and with no known sensory or motor impairments), who provided written informed consent before starting with the experiment. The evaluation was conducted in a quiet room, and the participants were seated in front of a screen displaying the evaluation questions. The evaluation data was collected between October and November 2023.

Based on previous analysis ^8,34,35^, 12 features were selected to evaluate the subjective perception of the surfaces covering tactile, visual, and auditory aspects of the surfaces. The features, which are described and listed in Table 2, were evaluated using a 1–10 scale.

**Table 2:** Features evaluated during the psychophysical evaluation.

**Fig. 6:**
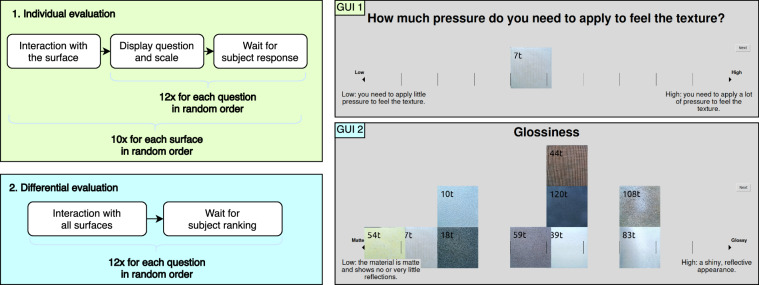
Psychophysical evaluation procedure. The evaluation was divided into two parts: Individual evaluation (Protocol 1 and GUI 1) and ranking evaluation (Protocol 2 and GUI 2). In the individual evaluation, participants were asked to rate the 12 features for each texture. In the ranking evaluation, participants ranked the textures for each feature.

The evaluation was divided into two parts: In the *Individual Evaluation*, participants were given a single texture at a time and were requested to slide along it with their index finger. They were then sequentially prompted with questions to rate the 12 features on a 1–10 scale (see GUI 1 in Fig. 6). In the *Ranking Evaluation*, participants were given all the textures at once and asked to rank them on the same scale for each question (see GUI 2 in Fig. 6). The order of the questions was randomized for each participant.

## Data Records

The data associated with this study is organized and archived in a public repository on figshare^36^, facilitating accessibility and reproducibility of the research findings. Each data record is detailed below, and the repository is cited accordingly.

### Data Accessibility

The dataset is stored in HDF5 (.h5) format, suitable for a wide range of computational environments. Additional metadata is provided in JSON format. The specific structure is detailed below. The dataset is organized into structured directories, each corresponding to a participant and subdivided by textures, themselves subdivided by trial.

### Physical data

The structure of the physical data recorded during the experiment is described in this section.

#### Naming convention

The following naming convention is used:

- subject_[ID]: ID of the participant (subject_0 to subject_9).
- texture_code: Code of the texture used during the trial (e.g., 7t).
- trial_type: Type of trial (std_0, std_1, std_2, cte_force_3, cte_speed_4).
- N: Number of data points recorded during the trial.
- f64: 64-bit floating-point number.
- int64: 64-bit integer.

#### Data structure

Each trial is stored in a separate file subject_[ID]_[texture_code]_[trial_type].h5 in HDF5 format, containing the following data:

- ft_sensor_data: Array of *N*_*ft*_ × 6f64 corresponding to the force and torque measured by the force/torque sensor: [*F*_*x*_, *F*_*y*_, *F*_*z*_, *T*_*x*_, *T*_*y*_, *T*_*z*_]. The forces are expressed in Newtons and the torques in Newton-meters.
- time_ft_sensor: Array of *N*_*ft*_ f64 timestamps (in second) of the data points.
- pos_data: Array of *N* × 8 f64 corresponding to the position of the two markers (*t*1 and *t*2) coordinates in the reference frame of the left camera (*cam*_1_): [${x}_{t1\in ca{m}_{1}}$, ${y}_{t1\in ca{m}_{1}}$, ${x}_{t2\in ca{m}_{1}}$, ${y}_{t2\in ca{m}_{1}}$]. The position is expressed in decimeters.
- time_pos: Array of *N*_*pos*_f64 timestamps (in s) of the data points.
- kistler_data: Array of *N*_*kistler*_ × 4 f64 corresponding to the vibrations measured by the two accelerometers and the two microphones: [*accelerometer*_*nail*_, *accelerometer*_*phalanx*_, *microphone*_*left*_, *microphone*_*right*_]. The accelerations are expressed in *g* and the sound in *kPa*.
- time_kistler: Array of *N*_*kistler*_ f64 timestamps of the data points.

#### Example

Here is an example of the structure of the data for the five trials of the participant 0, sliding on the texture 7t: In the directory subject_0/7t/ we find the following files:

- subject_0_7t_std_0.h5
- subject_0_7t_std_1.h5
- subject_0_7t_std_2.h5
- subject_0_7t_cte_speed_4.h5
- subject_0_7t_cte_force_5.h5

### Texture pictures

The texture images are stored in the directory called img. There is a folder for each texture containing the ten shots taken from the two cameras (twenty images per texture). The images are stored in JPEG format with a resolution of 3264 × 3264 pixels. Each image is named [texture_code]_[camera_code]_[shot_number].jpg with camera_code being either l or r for the left and right camera respectively.

### Questionnaire data

The questionnaire data is stored in a separate and unique JSON file. The file consists of the following structure:

- subject_[ID]: One entry per subject.
  - texture_eval: **Individual evaluation**
  - texture_code: One entry per texture.
    - question_code: One entry per question. It contains the integer value given by the participant.
  - questions_rank: **Ranking evaluation**
    - question_code: One entry per question.
      - Array: An array of 10 integers corresponding to the rank given by the participant for each texture.

### Data table

#### Physical data directory

The following Table 3 provides an overview of the physical data records:

Additionally the psychophysical questionnaire data is stored in a single JSON file. The files is named as *psychophysical_evaluation.json*.

**Table 3:** Overview of the physical data records from the 5 trials for each of the 10 textures and 10 participants.

Subject	Texture	Data Files
subject_0	7t	subject_0_7t_std_0.h5subject_0_7t_std_1.h5subject_0_7t_std_2.h5subject_0_7t_cte_speed_4.h5subject_0_7t_cte_force_5.h5
subject_0	10t	subject_0_10t_std_0.h5…subject_0_10t_cte_force_5.h5
subject_0	…	…
subject_0	120t	subject_0_120t_std_0.h5…subject_0_120t_cte_force_5.h5
subject_1	7t	subject_1_7t_std_0.h5…subject_1_7t_cte_force_5.h5
…	…	…
subject_9	120t	subject_9_120t_std_0.h5…subject_9_120t_cte_force_5.h5

#### Texture Images Directory

The images of each texture, independent of the participants, are stored in a different called img. Each texture has its own folder containing the images taken from the two cameras.

The images are named as [texture_code]_[camera_code]_[shot_number].jpg.

## Technical Validation

The dataset encompasses a wide range of tactile interactions, including friction-induced vibrations, load data and position data. It is further enriched with audio data, providing a comprehensive multimodal dataset for tactile analysis. To demonstrate the effectiveness of the dataset for capturing the complexities of tactile interactions, we have evaluated several combinations of modalities and features for surface classification. This provides a benchmark for future studies and demonstrates the importance of multimodal data for tactile analysis.

### Data preprocessing

To carry out efficient classification of the various surfaces, we extracted several features from the raw data, which could then be used for training machine learning models. Concerning *audio data*, it was shown in^37^ that Mel Frequency Cepstral Coefficient, Spectral roll-off, Chromagram, Pitch, Spectral Centroid, spectral bandwidth, and RMS energy work well for discriminating between different classes.

Compared to the more established fields of audio and image processing, the domain of *tactile data* analysis has fewer well-defined features, especially for analyzing friction-induced vibratory signals. Building upon our previous work^38^, we address this challenge by using audio-inspired features such as Mel-frequency cepstral coefficients(MFCCs), root mean square(RMS) energy, pitch, spectral centroid, spectral bandwidth, zero crossing rate(ZCR), and spectral roll-off. We hypothesize that these audio signal processing features can also effectively capture the characteristics of tactile signals. These features are described in Table 4.

**Table 4:** Audio-inspired features for tactile data processing.

Feature	Simplified Meaning
MFCCs	Mel-frequency cepstral coefficients: Extracts the power spectrum of bins of frequencies. The bins are spaced according to the MEL perceptual scale.
RMS Energy	Root mean square energy of the signal.
Pitch	The perceived frequency of the signal.
Spectral Centroid	The centre of mass of the frequency spectrum.
Spectral Bandwidth	The width of the frequency spectrum.
Zero Crossing Rate	The rate at which the signal changes sign.
Spectral Roll-off	The frequency below which a certain percentage of the signal’s energy is contained.

### Set of modalities

We focus our evaluation on the following set of modalities: *Audio*, using only the audio data captured by the microphones; *Acceleration*, using only friction-induced vibration data measured by the accelerometers; and *Tactile*, which includes a combination of load, position and friction-induced vibration data.

### Classifier

We used a Random Forest (RF) algorithm to classify the data, which is a popular ensemble learning method that combines multiple decision trees to improve classification performance. RF is well-suited for this large and multi-modal dataset, as it can handle high-dimensional data, is robust to overfitting, and is capable of handling imbalanced datasets, which is a common issue in tactile data classification. The RF classifier was trained on the extracted features from the tactile, audio, and acceleration data to classify the surfaces.

### Results

**Fig. 7:**
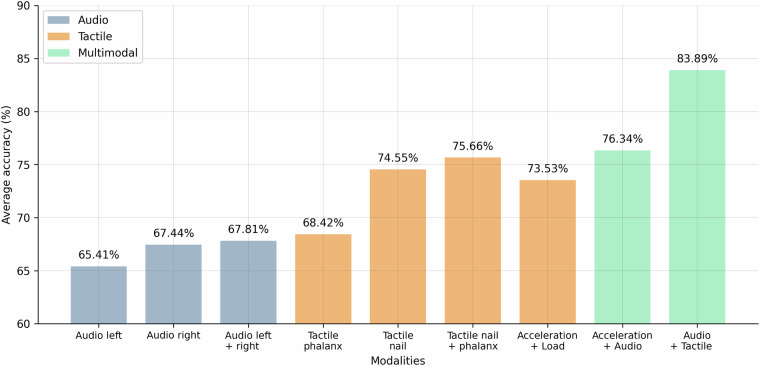
Classification Performance Comparison on Main Phase Data between Uni-modal and Multi-modal Models with Random Forest Classifier. The standard deviations are listed in Table 5.

The results of the classification are presented in Fig. 7 and Table 5. The models incorporating tactile data exhibited superior classification accuracy compared to those relying solely on audio or acceleration data. The combined tactile model achieved the highest accuracy and F1 score, highlighting the effectiveness of combining multiple tactile data sources.

**Table 5:** Classification Performance Metrics over the sets modalities.

Model	Average Accuracy	*σ*^2^	Average F1 Score	*σ*^2^
Audio left	0.6541	4.69 × 10^−7^	0.6552	4.42 × 10^−7^
Audio right	0.6744	2.96 × 10^−6^	0.6753	2.81 × 10^−6^
Audio left + right	0.6781	2.55 × 10^−6^	0.6728	1.03 × 10^−5^
Tactile phalanx	0.6842	4.87 × 10^−6^	0.6824	4.61 × 10^−6^
Tactile nail	0.7455	4.59 × 10^−6^	0.7441	4.62 × 10^−6^
Tactile nail + phalanx	0.7566	6.10 × 10^−6^	0.7550	5.72 × 10^−6^
Acceleration + Load	0.7353	4.02 × 10^−6^	0.7348	4.17 × 10^−6^
Acceleration + Audio	0.7634	1.07 × 10^−5^	0.7627	1.12 × 10^−5^
Audio + Tactile	0.8389	2.30 × 10^−6^	0.8387	2.17 × 10^−6^

While the *audio modality* achieved lower accuracy compared to the other modalities, it still resulted in more than 65% classification accuracy (much greater than the 10% chance level). The audio data from the right microphone performed slightly better than the left microphone, which could be due to slight differences in the microphone placement and the characteristics of external noise. Combining the two audio sources did not significantly improve the classification performance, indicating that the two audio sources do not provide complementary information.

The *tactile modality* outperformed the audio modality, achieving an average classification accuracy of 75.66% and an F1 score of 75.50%. The tactile data from the nail accelerometer performed significantly better than the phalanx accelerometer, indicating that the nail accelerometer captures more relevant information about the tactile interactions. This difference could be explained by the more rigid fixation of the nail accelerometer compared to the phalanx accelerometer. Nevertheless, the combination of the two tactile data sources significantly improved the classification performance, indicating that the two tactile data sources provide complementary information.

A key advantage of our dataset is the measurement of the fingertip position and speed during the tactile interactions: The classification performance using only the acceleration and load data was significantly lower (73.53% accuracy and 73.48% F1 score) than the full tactile data, indicating the importance of the position and speed information for tactile data classification.

The *combination of audio and tactile data achieved the highest classification performance*, with an average accuracy of 83.89% and an F1 score of 83.87%. This indicates that the audio and tactile data provide complementary information about the tactile interactions. Again, to show the importance of the position and speed information, we also evaluated the performance of the audio and tactile data without the position and speed information. The classification performance using only the acceleration and load data was significantly lower (76.34% accuracy and 76.27% F1 score) compared to the full audio and tactile data, indicating once more the importance of the position and speed information in tactile data classification.

### Questionnaire-based evaluation of textured surface perception

#### Individual texture evaluation

**Fig. 8:**
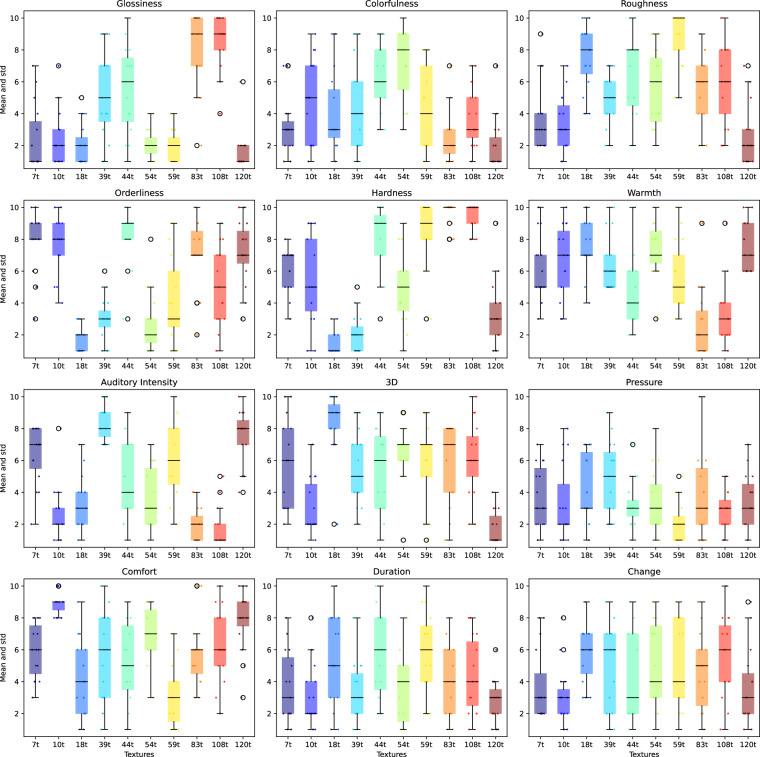
Distribution of the scores for each texture in the questionnaire.

In this first phase of the questionnaire, the textures were evaluated individually, requiring the participants to use their own reference to evaluate the textures. This participants’ subjective evaluation leads to a large variability in the ratings scores of the textures. For instance, texture “83t” (ribbed aluminium) received consistent scores for “hardness” (mean rating of 10, standard deviation < 0.1, with only two participants differing) while more subjective criteria such as “change” exhibited a larger variability reflecting differences in individual perception (Fig. 8).

#### Differential rating of texture characteristics

The differential evaluation of the textures for each questions enables us to evaluate the proximity of the textures in terms of their perceived characteristics, and the characteristics that are most relevant for the differentiation of the textures. To assess variations in texture ratings across questions, the ratings *v* = (*v*_1_, …*v*_12_) for the 12 questions were normalized across all textures {*t*} and participants {*p*} with

1 $${\overline{v}}_{i}(p,t)=\frac{{v}_{i}(p,t)-\min {v}_{i}}{\max {v}_{i}-\min {v}_{i}}\,,\quad \min {v}_{i}\equiv \mathop{\min }\limits_{p,t}\{{v}_{i}(p,t)\}\,,\quad \max {v}_{i}\equiv \mathop{\max }\limits_{p,t}\{{v}_{i}(p,t)\}$$

If $\min {v}_{i}=\max {v}_{i}$, ${\overline{v}}_{i}(p,t)\equiv 0.5$ to denote a neutral ranking. This avoids bias due to the rating’s range used in the response to a specific question. The normalized ratings were then used to create a vector representation for each texture, where each element of the vector corresponds to normalized ratings for each question. This vector representation can then be used to compare the textures in terms of the perceived characteristics.

*Principal Component Analysis* (PCA) was then applied to the normalized ratings to identify the principal components that capture the most variance in the dataset.

**Fig. 9:**
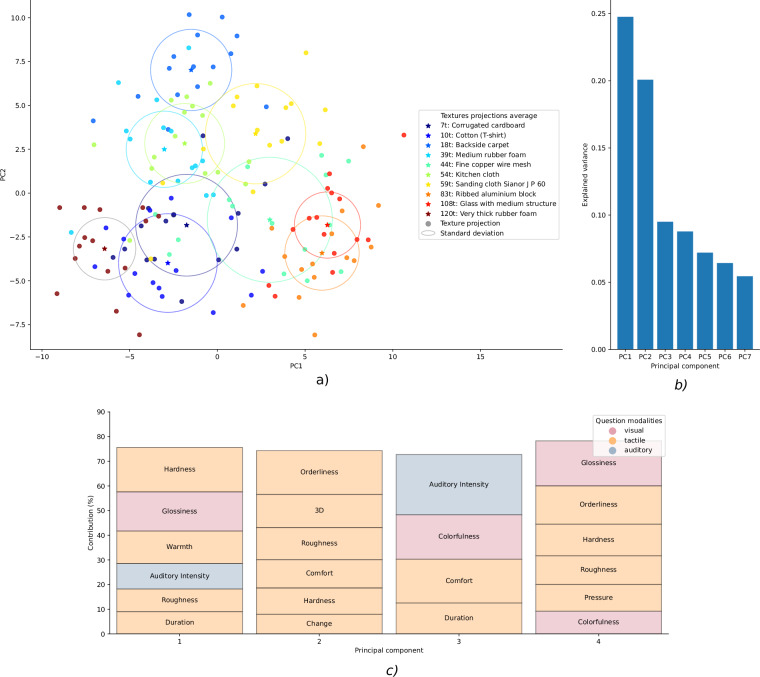
Principal Component Analysis of the normalized rankings of the textures. (**a**) Scatter plot of the textures in the reduced two-dimensional space defined by the first two principal components. To provide insight into the variability of the subjective evaluations within each texture, the mean and standard deviation of the samples in each texture group are visualized as a star marker and a circle, respectively (**b**) Percentage of variance explained by the first seven principal components. (**c**) Weights and modality of each question in the first four principal components.

The scatter plot in Fig. 9a was generated to visualize the distribution of textures in the reduced two-dimensional space where each texture is represented by a distinct color. The proximity of the textures in the reduced space gives insight into the perceived similarities and differences among the textures. When analyzing the principal components, the weights of each rating criteria indicate their contribution to the variance captured by the principal components. In Fig. 9c we see that the first principal component is strongly influenced by the rating score related to the texture ‘hardness’ while the second principal component is mainly influenced by the rating score related to the texture ‘orderliness’, ‘3D’ and ‘roughness’. This analysis provides insights into the factors that most influence the subjective texture differentiation.

However, the explained variance of the first two principal components is only 24% and 20% respectively (see Fig. 9b), indicating that the first two principal components capture only a small portion of the total variance in the dataset. This suggests that the subjective evaluations of the textures are influenced by a wide range of characteristics, and that the first two principal components may not capture the complexity of the texture evaluations well.

## Data Availability

The dataset described in the previous section “Data Record” and accompanying processing scripts are available on an online repository^36^. The repository includes a README.md file that provides detailed instructions on how to access and use the dataset.
